# Multiple sources and student engagement in the smart classroom: the mediating role of foreign language enjoyment under control-value theory

**DOI:** 10.3389/fpsyg.2026.1803799

**Published:** 2026-05-07

**Authors:** Jing Wu

**Affiliations:** School of International Communication, Wuhan Business University, Wuhan, China

**Keywords:** control-value theory, foreign language enjoyment, mediation, smart classroom, student engagement

## Abstract

Smart classroom environments offer enriched learning conditions for technology-enhanced foreign language learning, yet the affective mechanisms linking such environments to student engagement remain insufficiently understood. Grounded in Control-Value Theory (CVT), this study examines the psychological pathway through which smart classroom environments are associated with student engagement, with a particular focus on the mediating role of Foreign Language Enjoyment (FLE). A quantitative survey was conducted with 205 Chinese university English majors from three academic years. The study investigated the relationships among multiple sources in smart classrooms, FLE, and student engagement. Structural equation modeling (SEM) was used to test the hypothesized relationships, and mediation analysis was conducted to examine the indirect effects of FLE. The results showed that multiple sources in smart classrooms were positively associated with both FLE and student engagement. Mediation analysis further indicated that FLE substantially mediated the relationship between multiple sources and both behavioral engagement and cognitive engagement. These findings support the applicability of CVT in smart classroom contexts by showing that positive achievement emotions are closely linked to the association between environmental affordances and student engagement. The study suggests that the effectiveness of smart classroom environments depends not only on the availability of instructional support but also on their capacity to foster enjoyable learning experiences. Pedagogically, the findings highlight the importance of designing technology-enhanced learning environments that strengthen learners' sense of control, task value, and positive emotional involvement.

## 1 Introduction

Traditionally, student engagement in foreign language learning was viewed as a predictable response to stable instructional inputs within conventional, teacher-centered classrooms, where the Initiation-Response-Feedback (IRF) pattern dominated interaction and teachers controlled the pace, content, and direction of learning (Rehman et al., 2025; Wedin and Norlund Shaswar, 2019). In these settings, learning effort was reliably sustained through a single authority and a fixed syllabus, with students assuming largely passive roles that limited their communicative competence development and agentic participation

(Nguyen, 2024; Yang and Liu, 2024). However, the emergence of smart classroom environments has fundamentally disrupted these assumptions, reshaping the learning experience through “multiple sources” of input, characterized by diverse informational resources, interactive perspectives, and technology-enhanced blended instruction, thus challenging traditional, linear pedagogical models. Within this multi-source ecosystem, AI tools such as ChatGPT constitute an important technological affordance, enabling personalized support, enhancing writing performance, and enriching students' overall learning experiences (Farrokhnia et al., 2023; Perkins, 2023; Seetharaman, 2023; Tlili et al., 2023). These technologies can significantly foster Foreign Language Enjoyment (FLE), a positive achievement emotion characterized by the pleasure, interest, and satisfaction learners experience during foreign language learning (Dewaele and MacIntyre, 2014), as recently evidenced by Hwang et al. (2024). Simultaneously, as highlighted by Huang (2024), these tools facilitate multifaceted student engagement, drawing learners into a more active behavioral, emotional, and cognitive involvement in their educational pursuits (Fredricks et al., 2004).

At the same time, however, these affordances give rise to a potential paradox: the very features intended to enhance enjoyment and engagement may also create conditions conducive to distraction. On the one hand, emerging technologies have demonstrated substantial potential in improving student engagement. Specifically, AI-driven personalization and emotion recognition have been shown to positively reassess English as a Foreign Language (EFL) learners' affective and cognitive involvement (Ding and Xue, 2025), while AI-enhanced social-emotional learning frameworks further underpin the simultaneous cultivation of engagement and emotional well being (Zong and Yang, 2025). Extending beyond affective benefits, recent evidence suggests that AI-generated feedback catalyzes active cognitive processing, thereby facilitating adaptive engagement cycles across behavioral and emotional domains (Ge, 2026). Consequently, students in smart learning environments exhibit superior linguistic proficiency, a result directly attributable to such heightened, multidimensional engagement (Huang, 2024). On the other hand, these same affordances carry inherent risks that complicate their pedagogical value. The misuse of gamification in learning applications can inadvertently steer students away from their primary learning goals (Bueno Muñoz et al., 2023), creating digital distractions that significantly hinder performance (Pérez-Juárez et al., 2023). Furthermore, the rapid advancement of AI technologies has presented a systemic challenge to foreign language instruction, compelling educators to urgently reevaluate their pedagogical strategies, professional responsibilities, and digital competence (Yang and Li, 2024). Consequently, a gap remains in our understanding of the specific psychological mechanisms of how learners sustain effort in the technology-enhanced environment. Existing research suggests that technological affordances have an impact on reshaping internal psychological processes. For example, AI-supported learning has been shown to enhance learning confidence (Zhang et al., 2026), satisfy students' basic psychological needs for autonomy and competence (Hao et al., 2025), and improve self-efficacy, learning attitudes, and motivational orientation (Lee et al., 2022). When learners perceive themselves as competent, autonomous, and psychologically supported, they are more likely to invest sustained efforts. However, less attention has been given to the mediating role of FLE in the smart learning environment. In smart classrooms, where learners are simultaneously exposed to AI-mediated feedback, multimodal content, and peer interactions, FLE is likely to fluctuate dynamically in response to the richness and diversity of these multiple sources because it is a theoretically compelling mediator between smart classroom affordances and student engagement. There is, therefore, a critical need to investigate the emotional pathways that can successfully translate these multiple sources into sustained, meaningful learner engagement.

Control-Value Theory (CVT; Pekrun, 2006) provides the theoretical foundation for this study, as it explains how academic emotions emerge from learners' appraisals of perceived control and task value. In smart classroom environments, this multi-source instructional support may be associated with learners' control and value appraisals by shaping how manageable, meaningful, and engaging they perceive classroom learning activities to be. Guided by CVT, t the present study conceptualizes the multiple sources characteristic of smart classrooms as environmental antecedents that may be associated with student engagement through learners' control–value appraisals and the experience of FLE. By specifying this appraisal-based pathway, the study aims to clarify the affective mechanism that may underlie the relationship between resource-rich technological environments and sustained student engagement. In light of the shifting dynamics in modern language education, the significance of this research is twofold, offering both a theoretical advancement and an empirical expansion of the current literature. Theoretically, this study moves beyond fragmented analyses of smart classroom components by conceptualizing pedagogical, technological, and social-collaborative resources as a unified instructional ecosystem. By utilizing CVT as a lens, it elucidates the “black box” of the learner's internal state, providing a theoretically grounded explanation of how environmental inputs may be interpreted through learners' control and value appraisals and become associated with the FLE linked to engagement. Empirically, this research addresses the scarcity of evidence regarding the multidimensional nature of engagement in AI-enhanced contexts, specifically by quantifying the mediating role of emotion in driving deep cognitive investment over mere behavioral compliance.

## 2 Literature review

### 2.1 CVT

CVT provides an integrative social-cognitive framework that elucidates how individuals' cognitive appraisals of control and value serve as the primary proximal determinants of their emotional experiences (Pekrun, 2006). According to CVT, students' emotional experiences in academic settings are primarily shaped by their cognitive appraisals of control (i.e., perceived competence or agency over learning activities) and value (i.e., the perceived importance, usefulness, or intrinsic interest of the task). These control and value appraisals are influenced by environmental conditions, including instructional design, teacher support, feedback structures, and learning affordances (Pekrun and Stephens, 2010). In this sense, CVT explicitly positions the learning environment as a critical antecedent that shapes achievement emotions through its impact on learners' subjective evaluations.

Within this framework, achievement emotions arise when learners interpret academic tasks as both controllable and valuable (Pekrun et al., 2006). Among these emotions, enjoyment is conceptualized as a positive, activating, activity-focused emotion that emerges when students experience a sense of agency while attaching high intrinsic value to the learning activity (Pekrun, 2006; Pekrun and Stephens, 2010). In the second language acquisition research, FLE has emerged as a cornerstone of the “positive turn,” representing the pleasure and interest a student finds in the classroom environment (Dewaele and MacIntyre, 2014).

CVT further posits that achievement emotions serve as critical determinants of engagement and performance outcomes (Pekrun, 2006). Positive activating emotions such as enjoyment are theorized to broaden cognitive flexibility and build personal resilience, thereby acting as a primary engine for multidimensional student engagement (Shen, 2021; Zeng, 2021). Extensive empirical evidence suggests that FLE is significantly fostered by a synergy of external factors, such as enthusiasm and support (Dewaele and Li, 2021), and diverse instructional affordances, including interactive digital platforms (Hwang et al., 2024), AI-assisted tools (Dong et al., 2026), and varied pedagogical strategies (Pekrun et al., 2009).

Taken together, CVT provides a theoretical basis for examining how multiple sources in smart classrooms may shape students' engagement through the mediating role of foreign language enjoyment.

### 2.2 Multiple sources in smart classroom

A particularly innovative and transformative development in this educational revolution is the smart classroom, which integrates advanced technologies such as mobile devices, interactive whiteboards, and livestreaming equipment to redefine the boundaries of instruction (Macleod et al., 2018). Beyond mere hardware, this environment is defined by its functional capacity for real-time feedback and collaborative inquiry, providing learners with ubiquitous access to digital affordances that shift the classroom from a static space to a dynamic ecosystem (Hu et al., 2024). Central to the effectiveness of this environment is the integration of “multiple sources,” a multifaceted construct that goes beyond the mere use of technology to encompass a synergistic combination of diverse informational resources, multi-perspective interaction, and AI-supported blended instruction. These integrated sources of learning content drive instructional transformation by facilitating heightened interactivity (Raes et al., 2020), improved visualization (Al-Hunaiyyan et al., 2018), personalized learning (Barak et al., 2016), real-time feedback (Chiu and Churchill, 2015), and enhanced collaboration (Binkley et al., 2012). In the context of foreign language education, the impact of multiple sources is particularly salient; they provide the rich, multimodal linguistic input (Li, 2020) and authentic communicative affordances necessary (Satar, 2015) for developing proficiency. By leveraging varied interactional and blended tools, these sources create a supportive and dynamic environment that not only facilitates cognitive development but also serves as the environmental catalyst for positive emotional states and sustained student engagement (Elahi Shirvan et al., 2020; Mohammad Hosseini et al., 2022). Central to this contemporary pedagogical ecosystem is the integration of AI technology, which optimizes learning by satisfying core psychological needs for autonomy and competence, thereby bolstering self-efficacy and confidence (Hao et al., 2025; Zhang et al., 2026). Furthermore, this technological integration fosters a dynamic, multidimensional engagement system through personalized feedback and emotion recognition, which stimulates active cognitive processing and ultimately leads to superior academic performance in Second Language Acquisition (SLA; Ding and Xue, 2025; Zong and Yang, 2025).

### 2.3 FLE and multiple sources in smart classroom

FLE is conceptualized as a complex, multidimensional positive emotion that reflects the pleasure, interest, and sense of accomplishment learners experience within the second language classroom (Dewaele and MacIntyre, 2014). Studies indicate that FLE significantly influences language acquisition, as it correlates with motivation and learning strategies (Ranellucci et al., 2015), self-assessed academic effort (Fiedler and Beier, 2014), readiness to engage in the target language (Khajavy et al., 2017), and overall academic success (Jin and Zhang, 2018). When learners perceive activities as engaging, enjoyable, and pertinent, they are more inclined to participate actively, feel motivated, and interact effectively in the language acquisition process (Zeng, 2021). While various factors influence FLE, the role of environmental support is quintessential in sustaining the positive affect required for language acquisition (Elahi Shirvan et al., 2020). Extant empirical evidence robustly substantiates the pivotal role of supportive instructional environments in fostering positive affect. Specifically, perceived teacher support, manifested through autonomy, involvement, and structured guidance, has been shown to significantly predict FLE among EFL learners (Zhao and Yang, 2022), while interest-triggering activities and high-quality teacher-student relationships further catalyze these positive emotional states (Guo, 2021). Beyond general support, specific affective behaviors, such as teacher enthusiasm (Dewaele and Li, 2021) and the strategic use of humor (Solhi et al., 2023), serve as potent predictors of FLE.

Beyond the socio-interpersonal influence of teachers, the rapid evolution of smart classrooms has expanded the scope of such support to include a diverse array of technological affordances that further cultivate this positive emotional ecosystem.

By integrating online platforms, social media, and language learning apps, educators can significantly expand interactivity and exposure to authentic linguistic input (Gonzalez-Lloret and Ortega, 2014). This infusion of authentic resources is fundamental to establishing a supportive learning environment that prioritizes contextualized and engaging experiences (Mayer, 2014). Such a supportive atmosphere is further reinforced by the strategic use of multimedia, including videos, audio recordings, and interactive simulations, which caters to diverse learning preferences and renders complex language acquisition more accessible (Kukulska-Hulme and Shield, 2008). When these technological affordances are leveraged through learning management systems to provide structured, interactive skill development (Yang et al., 2013), they collectively shift the classroom from a traditional delivery model toward a highly stimulating ecosystem. Advancing this discourse into the AI era, Derakhshan (2025) provides critical empirical grounding for conceptualizing these AI-enhanced environments as multi-layered motivational ecosystems that dynamically shape learners' emotions and achievement-related orientations. Ultimately, this integrated environment serves to inherently sustain student motivation and foster deep, multidimensional engagement (Chen et al., 2023).

According to CVT, such comprehensive support is critical for fostering FLE because it directly enhances learners' appraisals of control over their learning activities and the subjective value they assign to those tasks (Pekrun, 2006).

### 2.4 FLE and student engagement

In the evolving landscape of SLA, student engagement is widely conceptualized as a multidimensional meta-construct encompassing learners' behavioral participation, cognitive investment, and emotional involvement in language learning (Fredricks et al., 2004). It reflects the dynamic relationship between learners and their educational environment and is increasingly regarded as a key predictor of academic persistence and long-term language development. Behavioral engagement correlates with the degree of students' active participation in learning, concerning students' efforts, commitment, and dedication (Sang and Hiver, 2021). Cognitive engagement is the mental effort that learners invest in their learning, which is typically demonstrated through their deliberate, focused, and sustained attention to academic tasks or objectives (Reeve, 2012). Emotional engagement encompasses the internal affective experiences and reactions triggered during language learning tasks (Fredricks et al., 2004).

Within foreign language learning specifically, engagement is indispensable; it facilitates the deep mental processing and sustained practice necessary for mastery while acting as the “engine” that converts potential interest into tangible linguistic achievement (Derakhshan and Azari Noughabi, 2024; Mercer and Dörnyei, 2020). Ultimately, robust engagement is the essential bridge between instructional input and successful acquisition, without which even the most advanced pedagogical tools remain largely ineffective (Çelik, 2025; Guo, 2021).

Existing literature identifies a range of major antecedents to engagement, broadly categorized into individual learner factors and contextual environmental affordances. Central to these environmental triggers is the role of instructional support, which provides the necessary scaffolding for student involvement through fundamental catalysts such as perceived teacher support (Zhao and Yang, 2022), teacher enthusiasm (Dewaele and Li, 2021), and the “safe harbor” created by positive teacher-student relationships (Li, 2023). Furthermore, the quality of the classroom social climate, characterized by student cohesiveness, task orientation, and teacher support, serves as a bedrock for both positive emotional experiences and multidimensional engagement (Mohammad Hosseini et al., 2022).

Recent shifts toward positive psychology underscore the paramount importance of FLE as the proximal determinant of this engagement, acting as the critical bridge between external support and active participation. As an “activating” emotion (Pekrun and Stephens, 2010), FLE functions by expanding a learner's cognitive flexibility and building personal resilience (Shen, 2021; Zeng, 2021), which in turn drives higher levels of student engagement (Botes et al., 2022; Guo, 2021;). This central role is further validated in modern learning contexts, where interactive affordances within digital platforms (Hwang et al., 2024) and AI-assisted instruction (Dong et al., 2026) are effective precisely because they foster the specific enjoyment and alleviate adverse emotional experiences (Derakhshan and Park, 2026) required to sustain learning engagement.

### 2.5 Multiple sources, FLE and student engagement

Within the framework of CVT, student engagement is conceptualized as a distal outcome of an affective-cognitive chain, where FLE acts as the primary internal conduit (Pekrun, 2006). Central to this process is the construct of “multiple sources,” which in a smart classroom functions as a tripartite system of environmental affordances encompassing instructional variety, technological support, and social-collaborative resources. Specifically, the instructional dimension, characterized by diversified task designs and blended activities, bolsters the subjective value of learning (Mayer, 2014), while the technological and social dimensions enhance perceived control through real-time feedback, relational scaffolding, and an individualized, interactive, and autonomy-supportive pedagogical framework (Derakhshan, 2025; Hwang et al., 2024; Wu et al., 2022).

The prioritization of these environmental affordances is empirically grounded in the finding that FLE is more significantly shaped by external contextual factors, such as teacher support and a conducive classroom environment, than by internal learner traits like language proficiency (Jiang and Dewaele, 2019). This highlights the decisive role of the smart classroom ecosystem as an environmental antecedent to learner affect. Crucially, the mediational capacity of FLE has been robustly demonstrated in prior SLA research, where it has been found to bridge the gap between internal traits and outcomes, such as mediating the link between grit and academic performance (Wei et al., 2019), as well as acting as the vital conduit through which teacher enthusiasm is converted into active social-behavioral engagement (Dewaele and Li, 2021).

Building upon these findings, the present study positions FLE as a key psychological mechanism through which multiple sources in smart classroom environments may be associated with sustained learner effort. By acting as a critical mediator, FLE converts the structural possibilities of the smart classroom into actual learning investment (Shen, 2021). Consequently, the efficacy of an integrated support system depends not merely on the presence of diverse digital or pedagogical tools, but on their collective capacity to foster a positive emotional state that catalyzes deep, multidimensional engagement.

## 3 Present study

Drawing on CVT, the present study conceptualizes “multiple sources” as a multifaceted environmental antecedent that directly optimizes the dual appraisals of control and value. In the smart classroom, these sources realize perceived control by providing learners with autonomous access to AI-mediated tools and personalized digital platforms, allowing them to regulate their learning pace and navigate linguistic challenges with greater agency. Simultaneously, the integration of authentic multimodal resources and collaborative social affordances enhances task value, rendering language acquisition more personally relevant and instrumentally rewarding. The “multiple sources” create the psychological foundation for FLE, which subsequently serves as the affective catalyst for heightened student engagement (Pekrun and Stephens, 2010).

While theoretical frameworks suggest a potent link between instructional support and learner affect, empirical evidence has remained fragmented, as researchers typically scrutinize environmental affordances in isolation rather than as a cohesive ecosystem. Current literature often bifurcates these inputs, with studies focusing exclusively on how social climate optimizes emotional states (Mohammad Hosseini et al., 2022) or how discrete instructional strategies drive positive affect (Guo, 2021). Even sophisticated ecological momentary assessments (Elahi Shirvan et al., 2020) tend to examine responses to isolated technology-mediated tasks, overlooking the influence of a unified support system. This study synthesizes these disparate affordances into the holistic construct of “multiple sources,” thereby unveiling the previously obscured mediational pathway through which technology-supported multiple sources of learning contents foster FLE to catalyze engagement. By positioning FLE as the primary psychological engine within this support-rich environment, this research provides a more comprehensive explanatory framework for understanding how the multiple sources of learning contents are converted into sustained learner efforts and engagement.

### 3.1 Research questions

1. To what extent do multiple sources predict FLE and the various dimensions of student engagement in a smart classroom environment?
2. Does FLE mediate the relationship between these multiple sources and the various dimensions of student engagement?

### 3.2 Research hypotheses

*H1*: Multiple sources will significantly and positively predict student engagement, encompassing its behavioral (H1a), cognitive (H1b), and emotional (H1c) dimensions.

*H2*: Multiple sources will have a significant positive effect on students' FLE.

*H3*: FLE will significantly and positively predict student behavioral (H3a), cognitive (H3b), and emotional (H3c) engagement.

*H4*: FLE will serve as a significant mediator in the relationship between multiple sources and student behavioral (H4a), cognitive (H4b), and emotional (H4c) engagement.

## 4 Method

### 4.1 Participants and procedure

The study involved English major students from a 4-year university situated in central China. To promote technology-enhanced education, this university has broadly integrated smart classroom technologies throughout various departments. Participants were recruited based on their enrollment in core courses utilizing this infrastructure, ensuring sustained engagement within a tech-supported learning context. The participants were briefed on the research objectives and informed that they could withdraw from the study at any time. We assured them that all survey data would remain confidential and be used solely for research purposes. Questionnaire links were distributed to participants *via* WeChat and QQ, resulting in a total of 205 valid responses. Table 1 provides an overview of the participants' demographic characteristics. The sample consisted of 205 students aged 17–22 years, including 38 males (18.5%) and 167 females (81.5%). With respect to academic standing, 32.7% were 1st-year students, 26.3% were in their 2nd year, and 41.0% were 3rd-year students. Fourth-year students were excluded from the sample due to their focus on graduation theses and absence from coursework during the semester.

**Table 1:** Demographic information of participants.

Variable	Category	*N*	%
Grade level	1st year	67	32.7
	2nd year	54	26.3
	3rd year	84	41.0
Gender	Female	167	18.5
	Male	38	81.5

### 4.2 Measures

#### 4.2.1 Multiple sources

Students' perceptions of multiple sources in smart classrooms were assessed using the Chinese adaptation of the *Preference Instrument of Smart Classroom Learning Environments* (Macleod et al., 2018). Specifically, in the present study, multiple sources was measured in terms of three aspects of smart classroom learning: diverse informational resources, multi-perspective interaction, and the integration of face-to-face and digitally supported instruction, including AI-assisted learning support. All items were rated on a 5-point Likert scale ranging from 1 (strongly disagree) to 5 (strongly agree). The internal consistency was acceptable (Cronbach's α = 0.828).

#### 4.2.2 Foreign language enjoyment

FLE was measured using the Chinese version of the *Foreign Language Enjoyment Scale* (Li et al., 2018), adapted for the smart classroom context. This dimension reflects learners' positive affect, sustained interest, perceived support from the learning environment, and enjoyment of English learning. Items were rated on a 5-point Likert scale (1 = strongly disagree, 5 = strongly agree). The scale showed strong internal consistency (Cronbach's α = 0.912).

#### 4.2.3 Student engagement

Student engagement was assessed with the Scale of *College Student Engagement* (Reeve and Tseng, 2011), adapted to the EFL smart classroom context. The scale comprised three dimensions: behavioral engagement (attentiveness and active participation during smart classroom activities), cognitive engagement (efforts to connect new knowledge with prior understanding and to employ integrative strategies for deeper comprehension) and emotional engagement (sense of curiosity, interest, and enjoyment toward learning content within the smart classroom environment). All items were rated on a 5-point Likert scale (1 = strongly disagree, 5 = strongly agree).

Preliminary analyses, however, indicated substantial conceptual and statistical overlap between emotional engagement and FLE. Theoretically, this overlap is understandable because emotional engagement typically reflects learners' positive affective involvement in learning, including curiosity, interest, and enjoyment (Reeve and Tseng, 2011), whereas FLE refers to the pleasure, interest, and satisfaction learners experience during the process of foreign language learning (Dewaele and MacIntyre, 2014). Statistically, the path from FLE to emotional engagement was extremely strong (β = 0.897), with FLE explaining 92.6% of the variance in emotional engagement (*R*^2^ = 0.926). In addition, retaining emotional engagement as a separate endogenous variable resulted in a weaker-fitting model (CFI = 0.945, TLI = 0.930, RMSEA = 0.098), whereas the more parsimonious model excluding emotional engagement showed improved fit (CFI = 0.962, TLI = 0.948, RMSEA = 0.091). Following SEM guidance that model respecification should be informed by both theoretical coherence and empirical redundancy, with attention to parsimony and interpretability (Kline, 2016), these results suggested considerable redundancy between FLE and emotional engagement in the present structural model and reduced the interpretability of FLE as the focal affective mediator. Accordingly, emotional engagement was not retained as an independent outcome variable in the final model, and the study focused on behavioral and cognitive engagement as the primary engagement outcomes. Reliability was satisfactory for the retained dimensions (Cronbach's α = 0.915 for behavioral engagement; Cronbach's α = 0.931 for cognitive engagement).

#### 4.2.4 Data analysis

Data were analyzed using SPSS 26.0 and AMOS 26.0. First, descriptive statistics and reliability tests were conducted. Subsequently, structural equation modeling (SEM) was employed to validate the measurement and structural models. Mediation effects were further examined through bootstrapping with 2,000 resamples; mediation was supported when the 95% confidence interval excluded zero.

## 5 Results

### 5.1 Descriptive statistics and correlations

In Table 2, descriptive statistics and correlations among the variables are presented. The mean scores for these constructs range from 3.888 to 4.220, suggesting generally positive perceptions among students. The standard deviations, ranging from 0.672 to 0.763, indicate moderate agreement and relatively low variability in the responses. The constructs' correlation coefficients indicated moderate to strong positive relationships, with *p*-values less than 0.01, ranging from 0.594 to 0.785.

**Table 2:** Correlations and descriptive statistics.

Variables	*M*	SD	MS	FLE	BE	CE
MS	4.220	0.672	1			
FLE	3.888	0.753	0.594^**^	1		
BE	3.909	0.747	0.599^**^	0.773^**^	1	
CE	3.930	0.763	0.622^**^	0.739^**^	0.785^**^	1

^**^The correlation is significant at the 0.01 level (two-tailed). MS, multiple sources; BE, behavioral engagement; CE, cognitive engagement; FLE, foreign language enjoyment.

### 5.2 Measurement model

#### 5.2.1 Convergent validity and internal reliability

Table 3 shows differences in internal reliability and convergent validity among the constructs. The Cronbach's alpha values, which assess internal consistency, range from 0.828 to 0.931, suggesting that most constructs have good reliability. The composite reliability (C.R.) values are all above 0.8, further supporting the internal consistency of the constructs. The average variance extracted (AVE) values are also above the recommended threshold of 0.5 for all constructs, indicating good convergent validity. The factor loadings for each item are substantial, with most loadings above 0.8, indicating those items strongly represent their respective latent constructs.

**Table 3:** Measurement model results.

Constructs	Items	Loadings	Cronbach's alpha	C.R.	AVE
MS	MS1	0.772	0.828	0.830	0.620
	MS2	0.828			
	MS3	0.761			
FLE	FLE1	0.835	0.912	0.910	0.771
	FLE2	0.899			
	FLE3	0.899			
BE	BE1	0.907	0.915	0.916	0.785
	BE2	0.898			
	BE3	0.852			
CE	CE1	0.873	0.931	0.932	0.820
	CE2	0.928			
	CE3	0.916			

#### 5.2.2 Discriminant validity

The discriminant validity was assessed to ensure that the items for each construct were unique and distinguishable from those of other constructs. As presented in Table 4, the square root of the AVE for each construct surpasses the correlations among constructs, with all inter-construct correlations remaining below 0.85, thus confirming the discriminant validity of the measurement model (Fornell and Larcker, 1981).

**Table 4:** Test of discriminant validity.

Construct	MS	FLE	BE	CE
MS	**0.787**			
FLE	0.594	**0.878**		
BE	0.773	0.599	**0.886**	
CE	0.739	0.622	0.785	**0.906**

The values in bold represent the square roots of AVE (Average Variance Extracted).

### 5.3 Structural model

#### 5.3.1 Model fit

As displayed in Table 5, the measurement model demonstrates a satisfactory fit with the data, with fit indices meeting established thresholds (Browne and Cudeck, 1992). The model fit metrics overall reflect statistical significance, supporting subsequent analyses.

**Table 5:** Fit indices.

Index	Reference value	Observed value
CMIN/DF	≤ 5	2.704
RMSEA	≤ 0.1	0.091
CFI	>0.8	0.962
TLI	>0.8	0.948
GFI	>0.8	0.911

#### 5.3.2 SEM path analysis results

As shown in Table 6, all hypothesized paths in the structural model were statistically significant, supporting the proposed model in the EFL context. Specifically, both the path from multiple sources to behavioral engagement (β = 0.211, *p* = 0.004) and the path from multiple sources to cognitive engagement (β = 0.304, *p* < 0.001) demonstrate positive influences, suggesting that the multiple sources enhance learners' engagement, supporting H1a and H1b. Furthermore, the positive influence of multiple sources on FLE (β = 0.685, *p* < 0.001) suggests that multiple sources enhance learners' FLE, supporting H2. The FLE exerts a strong positive effect on both behavioral engagement (β = 0.719, *p* < 0.001) and cognitive engagement (β = 0.606, *p* < 0.001), reinforcing the idea that FLE significantly boosts both behavioral and cognitive dimensions of engagement, supporting H3a and H3b. All critical ratios (C.R.) exceeded the recommended threshold of 1.96 (Kline, 2016), indicating the robustness of these relationships. Overall, these results underscore the importance of leveraging multiple sources and fostering enjoyment to enhance engagement in SLA.

**Table 6:** Hypothesis results.

Hypothesis	β	S.E.	C.R.	*p*
H1a: MS → BE	0.211	0.096	2.896	0.004
H1b: MS → CE	0.304	0.112	3.939	^***^
H2: MS → FLE	0.685	0.110	8.305	^***^
H3a: FLE → BE	0.719	0.080	8.880	^***^
H3b: FLE → CE	0.606	0.084	7.766	^***^

^***^p < 0.001 (two-tailed).

#### 5.3.3 Mediation effects

The findings, obtained through the bias-corrected percentile Bootstrap method with 2,000 resamples, are presented in Table 7, which examines the mediating effects of FLE. The indirect effect of multiple sources on behavioral engagement through FLE was 0.650, with a 95% CI excluding zero [BC CI = (0.430, 1.013), *p* = 0.002; PC CI = (0.412, 0.966), *p*= 0.003]. Similarly, the indirect effect of multiple sources on cognitive engagement *via* FLE was 0.600, with a 95% CI excluding zero [BC CI = (0.358, 0.967), *p* = 0.002; PC CI = (0.357, 0.964), *p* = 0.003], confirming its significance. The direct effect of multiple sources on cognitive engagement remained significant (β = 0.440, *p* < 0.05), whereas its direct effect on behavioral engagement was weaker (β = 0.279, *p* < 0.05). The total effects were substantial for both behavioral engagement (β = 0.929, *p* < 0.001) and cognitive engagement (β = 1.039, *p* < 0.001), underscoring the strong role of multiple sources in shaping engagement outcomes.

**Table 7:** Test of mediation effect.

Effect type	Point estimate	Product of efficient		Bootstrapping 2,000 times with 95% CI						
				BC			PC			
		SE	*Z*	Lower	Upper	*p*	Lower	Upper	*p*	Ratio
*Multiple sources→foreign language enjoyment→behavioral engagement*										
Indirect effect	0.650	0.170	3.824	0.430	1.013	0.002	0.412	0.966	0.003	30.0%
Direct effect	0.279	0.176	1.585	0.061	0.629	0.020	0.015	0.562	0.039	70.0%
Total effect	0.929	0.139	6.683	0.699	1.250	0.001	0.680	1.216	0.001	
*Multiple sources→foreign language enjoyment→cognitive engagement*										
Indirect effect	0.600	0.189	3.175	0.358	0.967	0.002	0.357	0.964	0.003	42.3%
Direct effect	0.440	0.218	2.018	0.113	0.819	0.014	0.044	0.757	0.029	57.7%
Total effect	1.039	0.131	7.931	0.834	1.373	0.000	0.811	1.324	0.001	

Finally, the proportion of the mediation effect was further examined. For cognitive engagement, 42.3% of the total effect was explained by the indirect pathway through FLE, while for behavioral engagement, 30.0% of the total effect was explained by the mediating mechanism. These results provide robust support for hypotheses H4a and H4b, highlighting that foreign language enjoyment plays a crucial mediating role in linking multiple sources with student engagement.

## 6 Discussion

The present research fills a gap in the previous research by examining the influence of integrated multiple sources on student engagement within the smart classroom. Rather than treating pedagogical variety (Li and Wang, 2022), technological affordances (Zong and Yang, 2025), and social-collaborative resources (Mohammad Hosseini et al., 2022) as separate, isolated variables, as much prior research has tended to do, this study conceptualizes them as a unified instructional ecosystem and suggests that their collective relationship with engagement is substantially mediated by FLE. This finding offers an important theoretical insight by suggesting that engagement in technology-enhanced environments may be closely linked to emotional appraisal processes, through which environmental stimuli are experienced affectively before being associated with learning behaviors and effort. It is precisely this appraisal-mediated logic that renders CVT (Pekrun, 2006) a theoretically appropriate framework for the present investigation. CVT is one of the few models in educational psychology that integrates environmental factors, cognitive appraisals, emotions, and achievement behaviors into a single, coherent explanatory chain, explicitly explaining the causal pathway from environmental inputs, through affective mediators, to downstream achievement outcomes (Pekrun, 2024). This integrative architecture is particularly apposite for smart classroom contexts, where especially AI-enhanced and blended environments simultaneously reshape learners' sense of control, by adaptive feedback (Cui and Zhang, 2025) and technological scaffolding (Derakhshan and Wei, 2026), and their perception of task value, by multimodal and interactive learning tasks (Wang et al., 2024), thereby activating the very appraisal mechanisms that CVT identifies as the proximal triggers of achievement emotions. By providing a unified account of how such environments may be related to both emotions and engagement, CVT is theoretically congruent with the design of the present study, in which FLE serves as the critical mediating variable linking the multiple sources of instructional support to learners' behavioral and cognitive engagement.

In alignment with the tenets of CVT, the findings pertaining to the first research question indicate that multiple sources, are positively associated with both FLE and student engagement. These results indicate that the smart classroom ecosystem acts as a potent environmental antecedent; specifically, the integration of interesting instructional materials, real-time digital feedback, and peer-teacher support fosters an emotionally resonant atmosphere that facilitates behavioral persistence and cognitive strategic engagement. As CVT posits, environmental variables influence achievement emotions by altering learners' appraisals of both their control over learning outcomes and the subjective value they assign to academic tasks (Pekrun, 2006, 2024). When learners are exposed to a technology-supported learning environment, their perceived control over learning is significantly enhanced. Their self-regulated learning is systematically fostered by AI-driven personalization and adaptive feedback (Sardi et al., 2025), while their self-efficacy is concurrently bolstered by the presence of robust teacher and technological support within AI-enhanced environments (Qiu, 2025). Together, these elements ensure that learners feel more capable and less overwhelmed, as the pedagogical support and AI affordances provide the necessary scaffolding for autonomous inquiry. Beyond the enhancement of control, these multiple sources significantly elevate learners' value appraisals of the educational task. The integration of AI-based chatbots and interactive tools fosters more positive learning attitudes and heightens intrinsic motivation by providing immediate, high-quality feedback that makes the learning process more meaningful (Lee et al., 2022). By transforming routine review tasks into engaging, interactive experiences, these technological affordances increase the subjective value students assign to their learning goals (Lee et al., 2022). Consequently, the simultaneous boost in perceived control and task value creates the optimal psychological conditions for FLE.

The finding that “multiple sources” significantly improve student engagement in the smart classroom is further supported by recent mixed-methods evidence from the study of AI-assisted language learning environments. The integration of diverse technological affordances, such as AI-based speaking partners and interactive platforms, serves as a primary driver for all three dimensions of engagement (Huang, 2024). Meanwhile, the AI feedback works as a critical mechanism for sustaining learner involvement. Further justifying this relationship, Ding and Xue (2025) highlight the specific role of AI-driven personalization and emotion recognition in navigating the affective challenges of digital environments. Their research indicates that when learners are supported by personalized technological resources, their “learning anxiety” is significantly mitigated, which in turn fosters higher levels of engagement. This suggests that the multiple sources in the present study do not just add variety but function as an emotional regulator. By tailoring input to the learner's specific level and personal needs, these sources reduce the cognitive and emotional “strain” that typically inhibits engagement in foreign language contexts (Ding and Xue, 2025).

From the perspective of CVT, the findings pertaining to the second research question indicate that FLE serves as a significant partial mediator between multiple sources and student engagement. The statistical results reveal that while environmental support directly stimulates engagement, a substantial portion of this effect, specifically 30.0% for behavioral engagement and 42.3% for cognitive engagement, is channeled through the activation of FLE. The robust mediation observed here aligns with the research by Elahi Shirvan et al. (2020) and Li (2023), suggesting that when students perceive high levels of pedagogical and technological support, their appraisals of control and value increase, thereby triggering the FLE necessary to sustain effortful learning. In the smart classroom, these supportive sources foster a “buffer zone” that heightens a learner's appraisals of control by reducing the strain that leads to anxiety (Ding and Xue, 2025) and appraisals of value by providing relevant, personalized input and support (Sardi et al., 2025). This pattern provides strong empirical support for the CVT proposition that environmental antecedents do not influence achievement outcomes in a vacuum; rather, they are filtered through and amplified by the learner's emotional experiences (Pekrun, 2006, 2024).

This mediation is further elucidated by Zong and Yang (2025), who argue that AI-enhanced social-emotional learning frameworks are transformative in fostering EFL students' emotional well being. By integrating AI tools that prioritize social-emotional needs, these sources establish a resilient psychological foundation that functions as an antecedent for heightened control and value appraisals, ultimately fostering deeper cognitive and behavioral engagement. This perspective is significantly extended by Ge (2026), whose “Adaptive Engagement Cycle” model demonstrates that AI-generated feedback serves as a critical stage where students actively interpret and emotionally react to their learning progress. The immediacy and quality of this feedback promote a sense of partnership in human-AI collaboration, shifting the learner from a passive recipient of support to an active agent in their own engagement cycle (Ge, 2026). This suggests that the “multiple sources” do not merely act as instructional aids; rather, they adjust the learner's internal appraisal system by providing the informational and emotional clarity needed to navigate complex learning tasks. By satisfying the psychological prerequisites of high control *via* adaptive feedback and high value *via* collaborative relevance, these sources systematically trigger FLE, through which sustained learner engagement and academic achievement are ultimately realized (Guo, 2021; Shen, 2021).

The fact that the mediation effect was stronger for cognitive engagement (42.3%) than for behavioral engagement (30.0%) suggests that while environmental support can directly compel students to “show up” and participate, the deeper, more strategic mental processing required for cognitive engagement is more heavily dependent on the genuine experience of FLE. This interpretation is consistent with Mohammad Hosseini et al. (2022), who emphasized that a favorable emotional climate is essential for higher-level cognitive engagement. It is further supported by Huang (2024) mixed-methods study of AI-assisted learning environments, which identified cognitive engagement as the strongest predictor of academic achievement. By fostering a state of flow and reducing the perceived difficulty of complex tasks, FLE allows students to transition from mere behavioral compliance to the deep, self-regulated processing necessary for mastery.

## 7 Implication

The findings of this study offer several critical theoretical and pedagogical implications for the field of second language acquisition in the digital age.

Theoretically, this study applies CVT to smart classroom contexts and provides empirical support for its core explanatory proposition: environmental conditions shape learners' control–value evaluations, which elicit achievement emotions and subsequently influence learning outcomes. In this study, multiple instructional sources functioned as environmental antecedents, while FLE emerged as a key mediating emotion linking these affordances to engagement. Specifically, FLE accounted for 42.3% of the variance in cognitive engagement and 30.0% in behavioral engagement. These findings verify the explanatory value of CVT in technology-enhanced language learning environments and highlight the importance of positive emotions as a central mechanism of sustained academic involvement.

Practically, the first suggestion is that teachers should design smart classroom instruction in ways that make multiple sources of learning support more conducive to students' perceptions of control and value. To strengthen students' sense of control, teachers can provide clear guidance, scaffold complex tasks into manageable steps, offer timely feedback through both human and technological channels, and give students more opportunities to make choices about learning pace, task sequence, or modes of participation. Such practices can help learners feel more competent, supported, and capable of succeeding in technology-enhanced environments. To enhance value, teachers should ensure that classroom activities are meaningful, interesting, and relevant to students' personal experiences, future goals, and real-life communication needs. For example, AI-supported discussions, collaborative problem-solving tasks, and interactive multimodal activities can increase students' sense that classroom learning is closely connected to real-life language use, supportive of students' personal development, and relevant to the demands and realities of the contemporary world. When multiple sources in smart classrooms are organized in ways that simultaneously enhance control and value, they are more likely to elicit FLE, which in turn sustains students' behavioral and cognitive engagement.

Second, this research offers practical implications for smart classroom design itself. Beyond equipping classrooms with technological infrastructure, schools should create learning spaces that help students feel both capable of succeeding and convinced that learning tasks are worth investing in. Flexible layouts, easy access to learning platforms, and supportive feedback systems can strengthen students' sense of control, while attractive classroom environments, meaningful learning scenarios, and personalized digital support can increase the perceived value of classroom activities. In this sense, smart classroom design should not be understood simply as a technical upgrade, but as the deliberate construction of a multi-source learning environment that fosters positive emotions and sustained engagement.

Third, this research provides suggestions for EFL teachers in the AI era. The booming Large Language Models appear to be transforming modern language education. Professional development initiatives should therefore equip educators to use AI strategically, enhancing learners' autonomy and task relevance while maintaining pedagogical support. By carefully balancing technological affordances with instructional scaffolding, teachers can transform diverse digital resources into sustained learner engagement.

## 8 Conclusion

In conclusion, the present study underscores the pivotal role of a source system in fostering learner engagement within the technology-rich landscape of the smart classroom. The findings demonstrate that enjoyment functions as a key psychological mechanism linking technology-enhanced learning environments and students' behavioral and cognitive engagement. By highlighting the importance of affective processes in digitally enriched classrooms, this study contributes to a more refined understanding of how complex instructional ecosystems may be associated with sustained learner participation. The results suggest that the effectiveness of smart classrooms lies not merely in technological presence but in how diverse learning resources are strategically integrated to support students' engagement.

Despite these contributions, several limitations must be acknowledged. First, the cross-sectional design limits the ability to make strong causal claims about the relationships among multiple sources, FLE, and student engagement. Although the proposed mediation pathway is theoretically grounded, the one-time measurement design does not establish temporal precedence, nor does it rule out reciprocal or reverse relationships among the variables. Future research could employ longitudinal, cross-lagged, or experimental designs to better capture the temporal and potentially bidirectional dynamics underlying these associations. Second, while the study focused on the overarching construct of “multiple sources,” further investigation is needed to determine the relative weight of each individual source, such as specific types of digital tools vs. teacher-led emotional support, in different cultural or institutional contexts. Additionally, as the study relied primarily on self-reported questionnaires, future studies might incorporate qualitative measures, such as classroom observations or semi-structured interviews, to provide a more nuanced understanding of the lived emotional experiences of learners in smart environments.

Future research should also explore the potential moderating roles of individual difference variables, such as learner grit or digital literacy, which may influence how students perceive and utilize the multiple sources available to them. Furthermore, investigating negative achievement emotions, such as foreign language classroom anxiety, alongside FLE would provide a more comprehensive view of the “emotional temperature” of the smart classroom. By continuing to explore these complex interactions, the field can better understand how to design instructional spaces that not only deliver content but also actively build the psychological resources and emotional resilience of language learners.

## Data Availability

The raw data supporting the conclusions of this article will be made available by the authors, without undue reservation.
